# Circulating levels of sclerostin but not DKK1 associate with laboratory parameters of CKD-MBD

**DOI:** 10.1371/journal.pone.0176411

**Published:** 2017-05-11

**Authors:** Geert J. Behets, Liesbeth Viaene, Björn Meijers, Frank Blocki, Vincent M. Brandenburg, Anja Verhulst, Patrick C. D’Haese, Pieter Evenepoel

**Affiliations:** 1 University of Antwerp, Dept. Biomedical Sciences, Laboratory of Pathophysiology, Wilrijk, Belgium; 2 KUL Leuven, Department of Immunology and Microbiology, Laboratory of Nephrology, Leuven, Belgium; 3 DiaSorin, Inc., Stillwater, Minnesota, United States of America; 4 University Hospital RWTH Aachen, Department of Cardiology, Aachen, Germany; University of California, Los Angeles, UNITED STATES

## Abstract

**Introduction:**

Mounting evidence indicates that a disturbed Wnt–β-catenin signaling may be involved in the pathogenesis of chronic kidney disease-mineral and bone and mineral disorder (CKD-MBD). Data on the impact of CKD on circulating levels of the Wnt antagonists sclerostin and Dickkopf related protein 1 (DKK1) and the relationship with laboratory parameters of CKD-MBD are incomplete.

**Methods:**

We analyzed serum sclerostin and DKK1 in 308 patients across the stages of chronic kidney disease (kDOQI stage 1–2 n = 41; CKD stage 3 n = 54; CKD stage 4–5 n = 54; hemodialysis n = 100; peritoneal dialysis n = 59) as well as in 49 healthy controls. We investigated associations with demographics, renal function, parameters of mineral metabolism including 25(OH) vitamin D, 1,25(OH)_2_ vitamin D, biointact fibroblast growth factor 23 (FGF23), and parathyroid hormone (PTH), and bone turnover markers.

**Results:**

Serum sclerostin, but not DKK1, increases in more advanced stages of CKD and associates with PTH, phosphate, and 1,25(OH)_2_ vitamin D concentrations. Bone turnover markers are highest in hemodialysis patients presenting the combination of high PTH with low sclerostin level. Serum DKK1 levels are lower in CKD patients than in controls and are not associated with laboratory parameters of mineral metabolism. Interestingly, a direct association between DKK1 and platelet count was observed.

**Conclusion:**

In CKD, serum levels of the Wnt inhibitors DKK1 and sclerostin are unrelated, indicating different sites of origin and/ or different regulatory mechanisms. Sclerostin, as opposed to DKK1, may qualify as a biomarker of CKD-MBD, particularly in dialysis patients. DKK1 serum levels, remarkably, correlate almost uniquely with blood platelet counts.

## Introduction

The (canonical) Wnt–β-catenin pathway is increasingly recognized to play an important role in bone [1] and vascular biology [2]. This pathway is tightly regulated by several antagonists, of which the soluble Wnt inhibitors Dickkopf related protein 1 (DKK1, 26kD) and especially sclerostin (28kD) have been studied most intensively. While sclerostin expression is largely limited to bone [3] and calcifying vascular tissue [4], DKK1 is expressed in a number of other tissues and cells including platelets, the prostate and the kidneys [5]. Since sclerostin and DKK1 not only exert local (paracrine) effects, but are also released in the systemic circulation, inhibition of Wnt signaling in distant tissues and organs can also occur. In SOST^-/-^ mice, for instance, it has been shown that kidney repair after unilateral urether obstruction is delayed [6] whilst in animal models of early CKD, incomplete recovery from acute kidney injury led to increased expression of Wnt inhibitors including DKK1 and sclerostin in the injured kidney and to increased levels in the systemic circulation [7]. Thus, DKK1 and sclerostin may also be involved in the many regulatory feedback loops that govern and fine-tune bone and mineral metabolism [8].

Circulating sclerostin levels increase with severity of chronic kidney disease (CKD) and are reported to reach levels that are 2 to 4-fold higher in patients with end stage renal disease as compared to individuals with normal renal function [9–15]. Data on circulating levels of DKK1 in CKD, conversely, are scarce and inconsistent with some investigators demonstrating increments already occurring in early stage CKD [16], while others showing levels in the normal range even in patients with advanced CKD [15, 17]. It is an ongoing debate to what extent sclerostin and DKK1 may serve as biomarkers of CKD-mineral and bone disorder (MBD) [18–20]. The purpose of this study was to evaluate circulating DKK1 and sclerostin levels in CKD and to describe for the first time the relationship between DKK1, sclerostin and prototypic laboratory parameters of mineral metabolism across stages of disease.

## Materials and methods

### Study population

The study population consisted of 308 prevalent CKD stage 1-5D patients and 49 controls. All patients were recruited from an ongoing observational study at the University Hospitals Leuven, Belgium, investigating uremic toxicity and bone and mineral metabolism in CKD patients (NCT 00441623). All patients were enrolled between February 2006 and July 2008. CKD stage 5D patients were treated either with thrice weekly conventional hemodialysis (n = 100) or peritoneal dialysis (PD, n = 59; continuous ambulatory PD: n = 30; Automated PD: n = 29). Dialysis adequacy was targeted in all patients according to the NKF K-DOQI guidelines. Controls, defined as individuals with no history of CKD and CKD-EPI estimated GFR > 60 ml/min 1.73 m^2^, were recruited from the dermatology outpatient clinic at the University Hospital Antwerp. All participants were 18 years of age or older and provided written informed consent. All studies were performed according to the Declaration of Helsinki, and approved by the Ethics Committees of the University Hospital Leuven and the University Hospital of Antwerp.

### Biochemical measurements

In all participants but HD patients, blood samples were collected in the morning (random, non-fasted). In HD patients, blood samples were collected before the mid-week dialysis session. After standard centrifugation, serum was aliquoted and stored at -80°C pending further analysis. Creatinine, hemoglobin, calcium, phosphate, C-reactive protein (CRP), total alkaline phosphatase (tAP), and cholesterol were all measured using standard laboratory techniques. Serum C-terminal cross-linked telopeptide (CTX-I) was measured using an electrochemiluminescence immunoassay (Roche Diagnostics, Switzerland). Albumin was measured using the bromocresol green method. Bone specific alkaline phosphatase (Bone ALP), calcidiol (25(OH) D), calcitriol (1,25(OH)_2_D) and PTH (N-TACT II) (i.e. a 2^nd^ generation PTH assay) were measured using a LIAISON XLautomated analyzer with the appropriate analyzer kits (DiaSorin, USA). Serum sclerostin (Biomedica, Austria), DKK1 (Biomedica, Austria), and biointact fibroblast growth factor 23 (FGF23, Kainos, Japan) were measured using ELISA kits according to the manufacturer’s instructions. As a complementary 3^rd^ generation PTH assay, whole (1–84) PTH (CAP PTH) was also measured using the Scantibodies CAP assay (USA). Detection limits of the various assays were: Bone ALP (0.1 μg/l); sclerostin (8.9 pmol/l); DKK1 (0.38 pmol/l); 25(OH)D (4.0 ng/ml); 1,25(OH)_2_D (< 2.0 pg/ml); CAP PTH (1.0 pg/ml); N-TACT PTH (1.7 pg/ml); FGF23 (3 pg/ml). Available reference values for healthy subjects are: sclerostin (11.9–47.9 pmol/l); DKK1 (47.7±20 pmol/l); calcitriol (25.1–66.1 pg/ml); CAP PTH (5–39 pg/ml); N-TACT PTH (14.5–87.1 pg/ml); FGF23 (8.2–54.3 pg/ml). All assays used report intra- and inter-assay variations below 15%. The eGFR was calculated using the CKD-EPI equation. Single pool Kt/V (spKt/V), a measure of dialysis efficacy was calculated using the second-generation logarithmic formula of Daugirdas [21]. Anuria was defined as a urine output <100 ml.

### Statistical analysis

Data are expressed as mean (standard deviation) for normally distributed variables or median (IQR) for non-normally distributed variables. Differences between groups were tested using parametric ANOVA, Kruskal-Wallis or chi-squared test as appropriate. Correlations between circulating levels of DKK1 and sclerostin and other variables were calculated by Spearman’s rank correlation coefficients. Multivariate linear regression analysis was performed including all univariately associated variables (p<0.2) to identify independent determinants of serum DKK1 and sclerostin. After excluding collinearity, the best subset of variables was selected by backward elimination on p <0.2. This subset was then subjected to a final elimination procedure on p <0.05. Inspection of residual plots assured that the a priori assumptions for linear regression were justified. For all statistical analysis, p-values less than 0.05 were considered significant. All statistical analyses were performed using SAS (version 9.3, the SAS institute, Cary, NC, USA).

## Results

### Demographics

**Fig 1:**
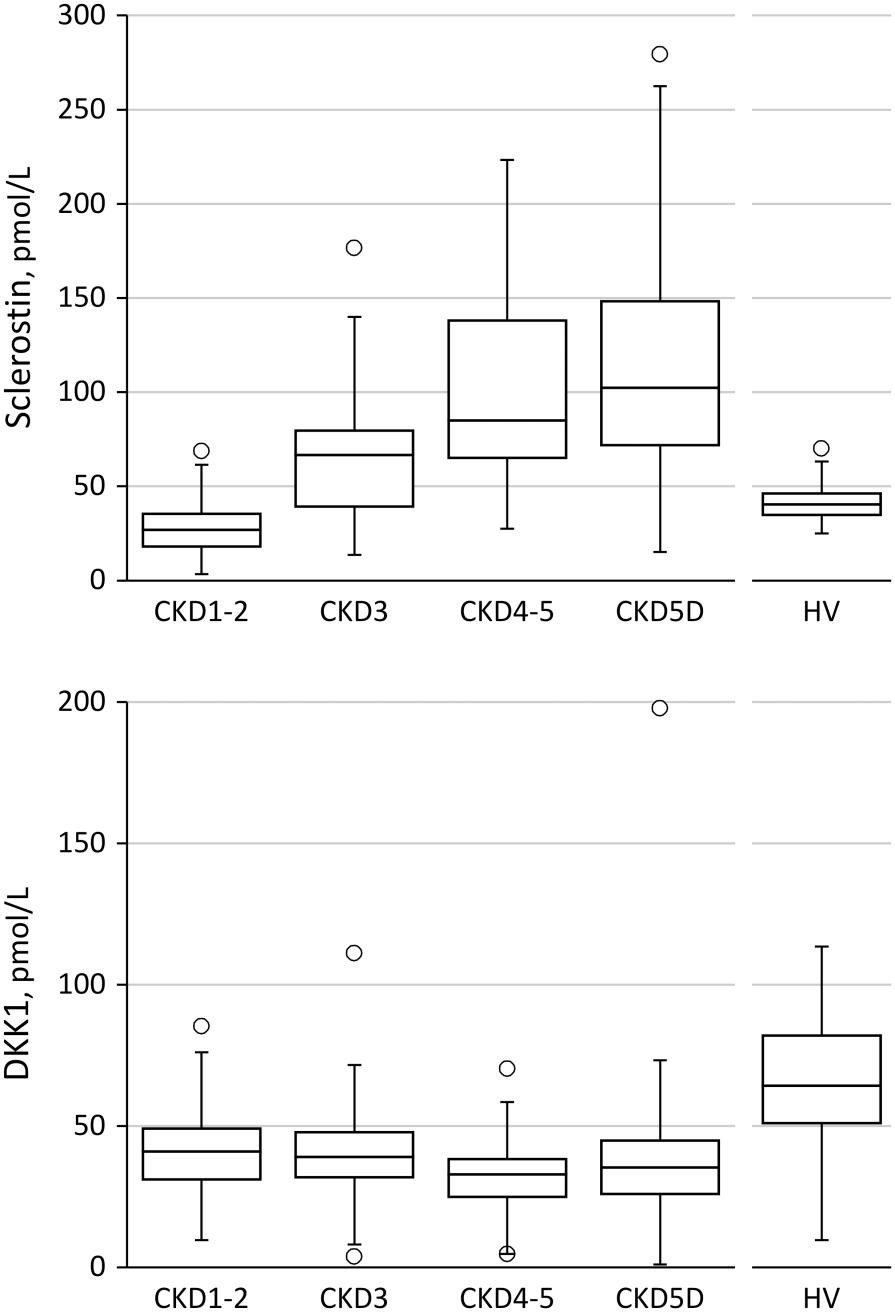
Serum sclerostin (A) and DKK1 (B) levels according to CKD stages and in healthy volunteers (HV).

Relevant clinical and biochemical characteristics of the healthy controls and CKD patients, categorized according to stage of disease, are summarized in Table 1. Serum sclerostin levels were higher and serum DKK1 levels were lower in CKD patients as compared to controls. Fig 1 shows serum sclerostin and DKK1 levels across CKD stages. In patients with chronic kidney disease treated with dialysis, sclerostin was approximately 2.5-fold higher than in non-CKD controls. Serum DKK1 levels, conversely were approximately 2-fold lower in dialysis patients as compared to non-CKD controls. S1 Fig shows temporal aspects of disordered mineral metabolism and sclerostin in CKD stage 1-5D.

**Table 1:** Demographics, biochemistry and therapy in healthy volunteers and CKD patients across stages.

	HV	CKD 1–2	CKD3	CKD 4–5	CKD5D	P (CKD)
N	49	41	54	54	159	
Age	50.88 ± 16.19	45.37 ± 14.69	64.07 ± 14.04	66.95 ± 11.47	63.47 ± 15.25	<0.0001
BMI	23.99 ± 33.64	25.84 ± 4.92	27.04 ± 5.60	27.59 ± 5.59	23.56 ± 4.14	<0.0001
Renal dx Diabetes (%)	-	0	1.9	1.9	17.6	<0.0001
Glomerular (%)	-	63.4	29.6	13.0	28.9	
Interstitial (%)	-	0	5.6	3.7	4.4	
Vascular (%)	-	2.4	7.4	20.4	13.8	
Cystic Heriditary (%)	-	9.8	11.1	14.8	4.4	
Miscellaneous, unknown (%)	-	24.4	44.4	46.3	30.8	
Male gender, %	41	32	56	70	60	0.002
CVD, %	-	14.6	31.5	44.4	40.9	0.21
DM, %	-	10	13	20	26	<0.05
Smoking, % (never/previous/current)	NA	62/22/16	66/20/14	45/37/18	46/37/18	0.14
Anti-platelet agents, %	0	10	35	52	51	<0.0001
Non-calcium PB, %	0	0	0	0	24.4	
Phosphate binder, %	0	12.2	14.8	35.2	86.5	<0.0001
Nutritional VitD, %	0	4.9	18.5	31.5	48.4	<0.0001
Active VitD, %	0	2.4	7.4	14.8	53.2	<0.0001
Calcimimetics, %	0	0	0	0	9	0.003
Bisphosphonates, %	0	5	15	6	4	<0.05
Hb, g/dL	-	14.1 ± 1.56	13.6 ± 1.5	12.5 ± 1.4	11.8 ± 1.3	<0.0001
Platelets, ×10^3^/mm^3^	-	280 ± 73	227 ± 68	208 ± 67	245 ± 83	<0.0001
Tchol, mg/dL	-	187 ± 32	181± 38	178 ± 35	163± 37	0.0009
CRP, mg/L	-	3.66 ± 6.15	3.55 ± 4.85	10.89 ± 26.93	8.30 ± 12.73	<0.0001
Albumin, g/L	-	44.79 ± 3.33	45.59 ± 2.11	44.48 ± 3.04	39.42 ± 3.78	<0.0001
Urea Nitrogen, mg/dL	-	33.5 ± 10,6	62.7± 21.2	110.3 ± 42.5	116.2± 32.4	<0.0001
Creatinine, mg/dL	0.94 ± 0.13	0.86 ± 0.14	1.49± 0.22	3.04 ± 1.43	7.25± 2.75	<0.0001
eGFR, mL/min 1.73m^2^	81.25± 14.45	79.75± 18.90	38.71± 7.67	19.07± 6.53	-	<0.0001
Ca, mg/dL	10.18± 1.16	9.18± 0.45	9.24± 0.35	9.09± 0.54	9.35± 0.73	<0.0001
Phos, mg/dL	4.02± 0.87	3.05± 0.58	3.13± 0.62	3.71± 0.83	4.51± 1.31	<0.0001
Bicarbonate, mmol/L	-	25.6 ±2.0	25.0 ±2.5	23.6 ± 2.6	25.0 ± 2.8	0.002
tAP, U/L	-	167.34 ± 45.77	182.23 ± 60.64	220.26 ± 111.55	254.25 ± 150.09	<0.0001
Bone ALP, μg/L	-	10.9 ± 4.7	12.1 ± 6.0	16.3 ± 14.5	18.7 ±15.0*	0.0003
CTX-I, ng/L	-	-	-	-	2539 ± 2392	-
25(OH)D, ng/mL	22.8 (15.7–27.5)	18.2 (12.1–26.2)	17.3 (13.3–25.0)	14.6 (10.9–19.3)	14.8 (10.3–20.6)*	0.08
1,25(OH)_2_D, pg/mL	-	82.0 (47.2–111.1)	54.0 (29.1–92.0)	30.5 (23.0–43.6)	-	<0.0001
N-TACT PTH, pg/mL	19.7 (14.0–24.7)	18.4 (11.4–27.6)	29.9 (22.6–44.3)	62.95 (46.0–137.0)	80.95 (45.2–154.0) *	<0.0001
CAP PTH, pg/mL	45.7 (39.2–63.3)	20.4 (12.4–38.0)	32.1 (25.4–47.6)	77.6 (48.7–123.4)	166.5 (67.7–341.4)	<0.0001
Sclerostin, pmol/L	40.5 (34.8–46.2)	27.0 (18.1–35.45)	66.7 (39.1–79.6)	85.0 (63.8–138.2)	102.4 (72.0–148.9)	<0.0001
DKK1, pmol/L	64.3 (51.0–82.0)	41.0 (31.0–49.1)	39.1 (31.9–47.9)	32.9 (24.9–38.4)	35.30 (25.6–45.0)	0.02
FGF23, ng/L	41.54 (35.1–49.1)	35.9 (30.3–45.9)	65.2 (49.1–91.4)	155.7 (93.0–279.2)	3725.0 (824.4–9963.1)	<0.0001

### Serum sclerostin in CKD

In CKD patients not yet on dialysis (S1 Table), male gender, history of CVD, higher age, phosphate, FGF23, PTH (both assays), and lower eGFR, bicarbonate, calcitriol, blood platelets all were significantly associated with higher serum sclerostin levels. In multivariable analysis, only gender, age, eGFR and calcitriol independently associated with serum sclerostin levels, explaining 54% of its variability (p<0.0001).

In CKD stage 5D patients (S2 Table), male gender, hemodialysis as modality, history of cardiovascular disease (CVD), higher age and phosphate and lower bicarbonate, PTH (both assays), residual renal function, and blood platelets all were significantly associated with higher serum sclerostin levels. In multivariate analysis, only gender, phosphate and N TACT PTH independently associated with serum sclerostin levels, explaining 16% of its variability (p<0.0001).

### Serum DKK1 in CKD

**Fig 2:**
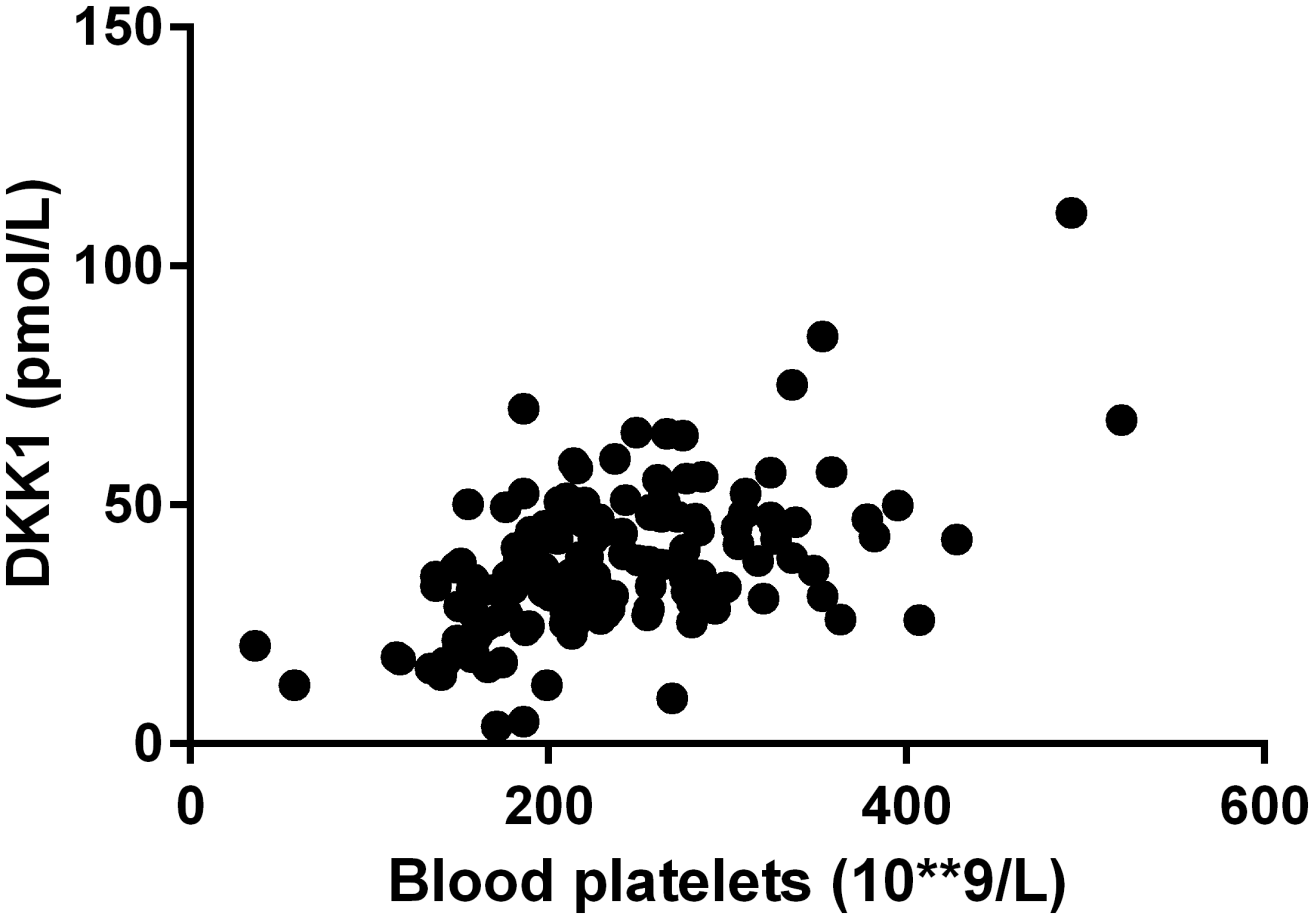
Correlation between blood platelet count and serum DKK levels in CKD patients not yet on dialysis (R^2^ = 0.28, p<0.0001, Spearman).

In CKD patients not yet on dialysis (S1 Table), higher bicarbonate, eGFR, blood platelets, and lower FGF23 all are significantly associated with higher serum DKK1 levels. In multivariable analysis, only bicarbonate and blood platelets independently associated with serum DKK1 levels, explaining 25% of its variability (p<0.0001). Fig 2 shows the correlation between blood platelet count and serum DKK1 concentration in CKD patients not yet on dialysis.

In CKD stage 5D patients (S2 Table), higher calcium, CRP, and blood platelets and lower PTH all were significantly associated with higher serum DKK1 levels. In multivariable analysis, only calcium and blood platelets independently associated with serum DKK1 levels, explaining 14% of its variability (p<0.0001). Of note, no association was observed between serum DKK1 levels and use of antiplatelet agents. Serum sclerostin and DKK1 levels did not correlate, neither in the overall cohort nor in subgroups.

### Sclerostin, DKK1 and bone turnover biomarkers in HD patients

**Fig 3:**
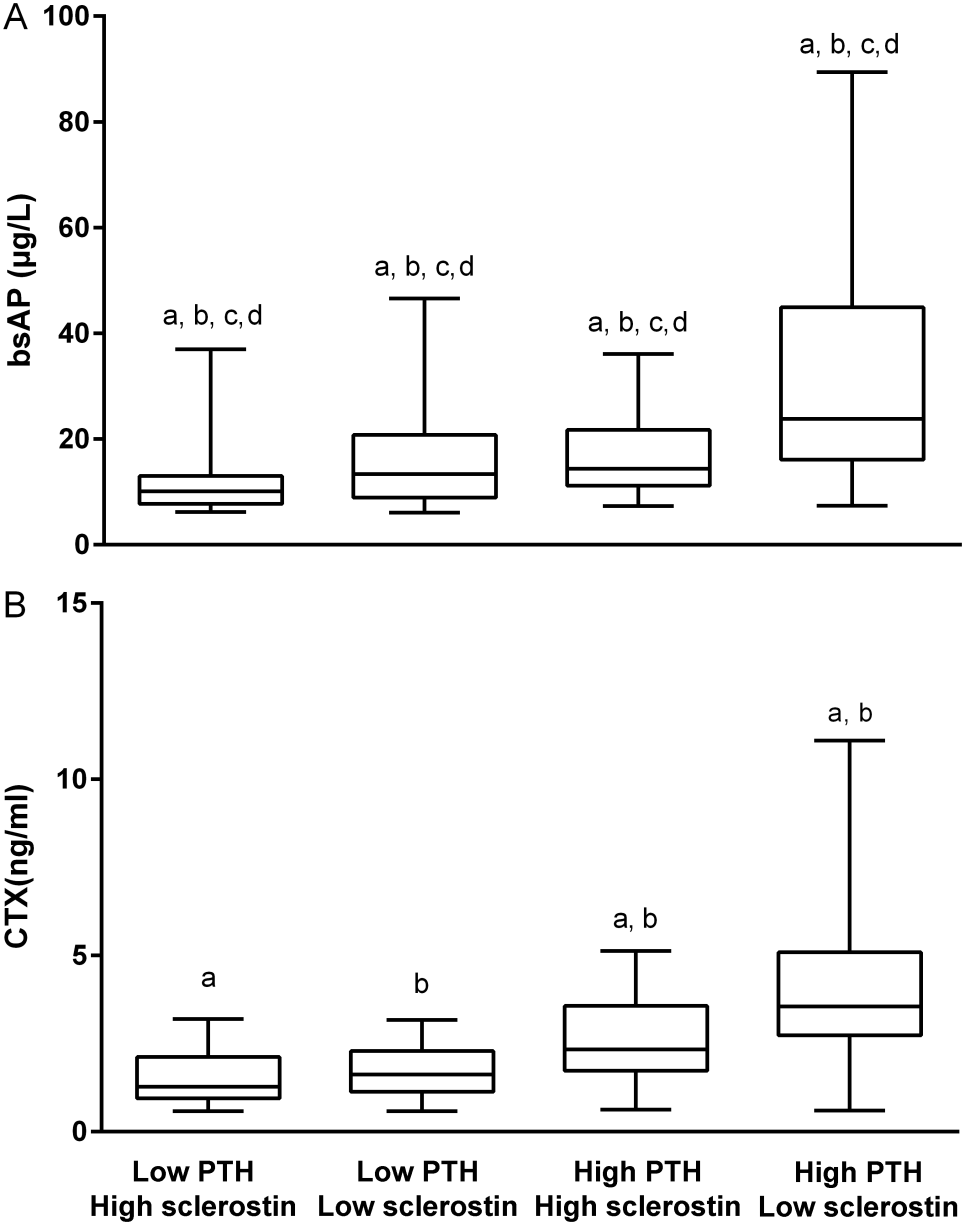
Bone-specific alkaline phosphatase level (Bone ALP) (A) and C-terminal telopeptide of collagen type 1 (CTX-I) (B), categorized according to PTH and sclerostin levels above [high] or below [low] the median. Groups with same indices differ significantly.

As eGFR turned out to be a major confounder of the relationship between Wnt inhibitors, in particular sclerostin, and bone metabolism, we further investigated the correlation of sclerostin, DKK1, laboratory parameters of mineral metabolism and markers of bone formation (Bone ALP) and resorption (CTX-I) in hemodialysis patients only (Table 2). PTH (both assays) strongly and directly correlated with Bone ALP and CTX-I. Sclerostin, as opposed to DKK1, tended (p ≤ 0.1) to correlate inversely with bone formation and resorption. Fig 3 shows the mean Bone ALP and CTX-I levels in patients categorized according to PTH and sclerostin levels above or below the median. Patients with high (above the median) PTH in combination with low sclerostin (below the median) had the highest Bone ALP and CTX-I levels. In regression analyses, low sclerostin independently associated with high Bone ALP levels (but not CTX-I levels), independent of PTH.

**Table 2:** Spearman correlation matrix HD patients.

	***Ca***	***Phos***	***N-TACT PTH***	***CAP PTH***	***Sclerostin***	***DKK1***	***FGF23***	***Bone ALP***	***CTX-I***
***Ca***	1	0.05	-0.04	0.05	-0.05	0.15	0.36^Y^	0.08	-0.03
***Phos***		1	0.37 ^Y^	0.33^X^	0.16	-0.06	0.57 ^Z^	0.06	0.41 ^Z^
***N-TACT PTH***			1	0.99^Z^	-0.19 ^(p = 0.07)^	-0.10	0.32 ^Y^	0.57 ^Z^	0.73 ^Z^
***CAP PTH***				1	-0.20 ^(p = 0.06)^	-0.09	0.31 ^Y^	0.63 ^Z^	0.70 ^Z^
***Sclerostin***					1	-0.05	0.06	-0.18^(p = 0.10)^	-0.18 ^(p = 0.08)^
***DKK1***						1	-0.02	-0.13	-0.09
***FGF23***							1	0.03	0.32 ^X^
***Bone ALP***								1	0.58 ^Z^
***CTX-I***									1

^X^: p < 0.05;

^Y^: p < 0.01;

^Z^: p < 0.001

## Discussion

A first finding of the present study is that circulating levels of sclerostin, as opposed to DKK1, increase with severity of CKD, reaching levels that are 2–3 fold higher than in non-CKD controls.

This increase is likely the result of an increased production of sclerostin, since a recent clinical study in 120 patients with CKD stage 1–5 showed an increased rather than a decreased absolute and fractional urinary excretion of sclerostin with declining kidney function [10]. Furthermore, in jck mouse, a genetic model of polycystic kidney disease that exhibits progressive renal disease, a transient increase in bone sclerostin was observed already in early stage disease [22]. Till today, it is not clear which mechanism underlies signaling to the skeleton to increase the production of sclerostin in the setting of CKD.

Besides an association with kidney function, we observed significant associations between circulating sclerostin levels and various laboratory parameters of CKD-MBD. These associations were most pronounced in CKD stage 5D patients. Most probably, the overwhelming impact of eGFR on circulating sclerostin levels obscured associations with laboratory parameters of mineral metabolism in CKD patients not yet on dialysis.

Serum 1,25(OH)_2_D levels negatively associated with circulating sclerostin levels in CKD patients not yet on dialysis, independent of eGFR, 25(OH)D, PTH and FGF23. This observation is in line with recent experimental evidence by Ryan *et al*. [8]. These investigators showed increased 25-hydroxyvitamin D 1α-hydroxylase cytochrome P450 (*cyp27B1*) mRNA in kidneys of *SOST* KO mice as compared to their wild types. Moreover, treatment of cultured proximal tubule cells with mouse recombinant sclerostin decreased *cyp27B1* mRNA transcripts. Whether vitamin D, reciprocally, affects *SOST* expression and circulating sclerostin levels remains to be investigated. Of note, circulating sclerostin in the present study did not differ between patients on and off therapy with active and/or nutritional vitamin D (data not shown).

In agreement with previous studies, we observed a positive and independent association between serum phosphate and sclerostin levels [9, 13, 14]. Additional studies are required to unravel the underlying regulatory mechanisms. In this context it is worth to be mentioned that cross-sectional studies investigating the association between sclerostin and FGF23, yielded conflicting results with some studies (including present study) reporting no association [11] and other studies observing a positive association [23].

In agreement with previous studies [11, 24], we observed an inverse relationship between sclerostin and PTH concentrations in dialysis patients. These data confirm and extend clinical observations in patients with non-renal parathyroid disorders [25–27] and are consistent with experimental data demonstrating downregulation of *SOST* by PTH [28]. Of note, high sclerostin levels coexist with high PTH levels in patients with advanced CKD. This observation suggests skeletal resistance to the action of PTH [29–31], similar to FGF23 resistance explaining the coexistence of high FGF23 and PTH levels in advanced stage CKD [32]. Of note, gender, serum phosphate and PTH levels determined only 16% of the variability of sclerostin levels in CKD stage 5D patients, implying that many other systemic and local determinants remain to be identified.

Consistent with the biological effects of sclerostin on bone, we observed an inverse relationship between serum sclerostin and Bone ALP, a bone formation marker, and between serum sclerostin and CTX-I, a bone resorption marker. As such, our data in HD patients confirm and extend previous clinical data [11, 12, 33]. Of interest, levels of the bone turnover biomarkers were highest in patients with high PTH levels in combination with low sclerostin levels.

Contrary to sclerostin, circulating DKK1 levels were not or only marginally associated with kidney function and parameters of mineral metabolism. Previous studies investigating the association between DKK1 and kidney function yielded conflicting results with some investigators observing unaltered [15, 17], while others reported increased [16] DKK1 levels in CKD. Of note, serum levels of DKK1 and sclerostin were unrelated in the present study, pointing to different origin and different regulatory mechanisms.

Importantly, the expression of sclerostin and DKK1 is not restricted to bone. Substantial evidence indicates that platelets may be a major source of circulating levels of DKK1. Moreover, it has been demonstrated that during clotting *ex vivo*, DKK1 is released from platelets to a significant but variable extent [34]. In a cohort of healthy volunteers, levels of DKK1 levels were 2.7-fold higher in serum samples as compared to plasma samples [34]. Whether the degree of this *ex vivo* release is a random phenomenon or relates to the *in vivo* activation state of the platelets, as suggested by some investigators, is a matter of ongoing discussion [35]. Of interest, in the present study, only platelet count and calcium, playing a crucial role in platelet activation [36], were found to be independently associated with serum DKK1 levels in dialysis patients and CKD patients not yet on dialysis. As opposed to others [37], we failed to demonstrate lower DKK1 levels in CKD patients receiving antiplatelet drugs compared with those not on antiplatelet therapy.

Significant but often complex associations have been reported between circulating levels of sclerostin and DKK1 and indices of vascular health [13, 15, 20, 38, 39]. Both sclerostin and DKK1 may be considered mediators and markers of cardiovascular disease (CVD). In the present study, and opposite to recent studies in non-CKD patients [35, 40], we failed to find higher circulating DKK1 and sclerostin levels in CKD patients with CVD as compared to counterparts free of CVD.

In conclusion, serum levels of the Wnt inhibitors DKK1 and sclerostin are unrelated in CKD, reflecting a different origin and different regulatory mechanisms. Sclerostin, as opposed to DKK1, may qualify as a biomarker of CKD-MBD, particularly in dialysis patients. DKK1 serum levels mainly relate to platelet count and/or activity. Additional experimental and clinical studies are required to elucidate the (path)physiological role of circulating sclerostin and DKK1 in bone disease and beyond. This information is mandatory as anti-sclerostin and anti-DKK1 monoclonal antibodies emerge as very promising pharmaceuticals in the treatment of osteoporosis.

## Supporting information

S1 Fig Sclerostin and mineral metabolism markers according to CKD stage. (TIF)

S1 Table Linear regression analysis with Ln sclerostin and ln DKK1 as dependent variable in CKD patients, not yet in dialysis. (DOCX)

S2 Table Linear regression analysis with Ln sclerostin and ln DKK1 as dependent variable in CKD patients on maintenance hemodialysis. (DOCX)
