# Supplementary figures and images for: Circulating levels of sclerostin but not DKK1 associate with laboratory parameters of CKD-MBD
Source: PLoS One. 2017 May 11;12(5):e0176411. doi: 10.1371/journal.pone.0176411 (PMC5426702; doi:10.1371/journal.pone.0176411)

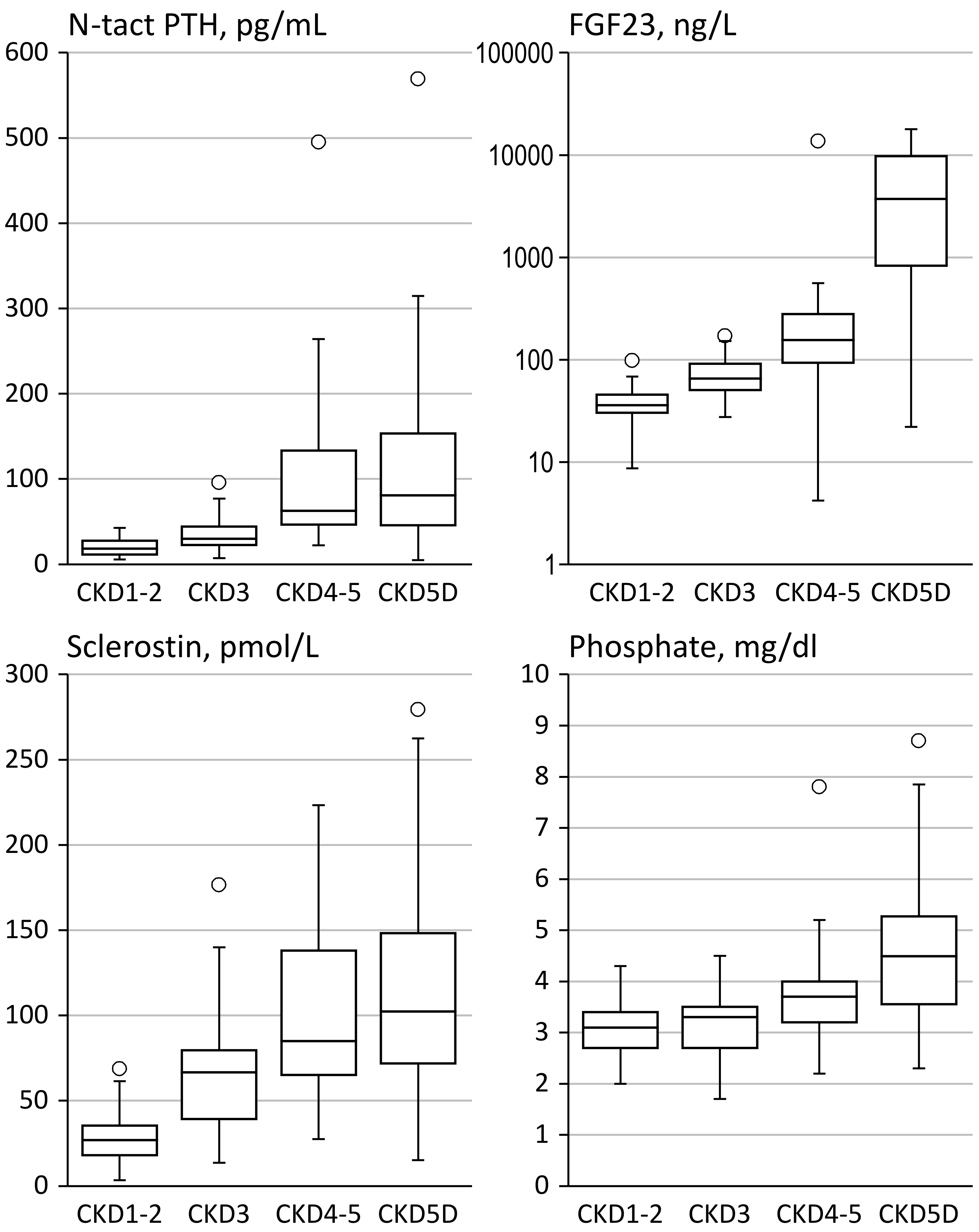

Supplement: S1 Fig — (TIF) [file pone.0176411.s001.tif]
