# Supplementary material for: Circulating levels of sclerostin but not DKK1 associate with laboratory parameters of CKD-MBD
Source: PLoS One. 2017 May 11;12(5):e0176411. doi: 10.1371/journal.pone.0176411 (PMC5426702; doi:10.1371/journal.pone.0176411)
Supplement: S1 Table — (DOCX) [file pone.0176411.s002.docx]

**Supplementary Table 1**: Linear regression analysis with Ln sclerostin and ln DKK1as dependent variable in CKD patients, not yet in dialysis

|  |  | **Ln sclerostin** | | | | | | **Ln DKK1** | | | | | |
| --- | --- | --- | --- | --- | --- | --- | --- | --- | --- | --- | --- | --- | --- |
|  |  | **Univariate** | | | **Multivariate (R²=0.54)** | | | **Univariate** | | | **Multivariate (R²=0.25)** | | |
| **parameter** | **unit** | B | SE | p | B | SE | p | B | SE | p | β | SE | p |
| **Age** | **yr** | 0.01 | 0.001 | <0.0001 | 0.004 | 0.001 | 0.003 | -0.0008 | 0.001 | 0.5 |  |  |  |
| **BMI** | **Kg/m²** | 0.002 | 0.005 | 0.6 |  |  |  | 0.005 | 0.003 | 0.1 |  |  |  |
| **Gender** | **M 0, F 1** | -0.3 | 0.05 | <0.0001 | -0.12 | 0.04 | 0.003 | 0.03 | 0.03 | 1 |  |  |  |
| **Diabetes** | **No:0; Yes:1** | 0.08 | 0.08 | 0.3 |  |  |  | 0.03 | 0.05 | 0.5 |  |  |  |
| **eGFR** | **mL/min 1.73m²** | -0.008 | 0.0007 | <0.0001 | -0.005 | 0.001 | <0.0001 | 0.002 | 0.0006 | 0.01 |  |  |  |
| **Ca** | **mg/dL** | -0.03 | 0.06 | 0.6 |  |  |  | -0.01 | 0.04 | 0.8 |  |  |  |
| **Phos** | **mg/dL** | 0.1 | 0.03 | 0.001 |  |  |  | -0.04 | 0.02 | 0.08 |  |  |  |
| **Bicarbonate** | **mmol/L** | -0.02 | 0.01 | 0.03 |  |  |  | 0.02 | 0.006 | 0.02 | 0.01 | 0.006 | 0.01 |
| **25(OH)D** | **ln, ng/L** | 0.006 | 0.05 | 0.9 |  |  |  | -0.05 | 0.03 | 0.1 |  |  |  |
| **1.25(OH)2D** | **Ln, pg/L** | -0.21 | 0.03 | <0.0001 | -0.07 | 0.02 | 0.02 | 0.02 | 0.02 | 0.5 |  |  |  |
| **N TACT PTH** | **ln, pg/mL** | 0.13 | 0.03 | <0.0001 |  |  |  | -0.02 | 0.02 | 0.2 |  |  |  |
| **CAP PTH** | **Ln, pg/ml** | 0.17 | 0.03 | <0.0001 |  |  |  |  |  |  |  |  |  |
| **FGF23** | **ln, mg/dL** | 0.15 | 0.02 | <0.0001 |  |  |  | -0.04 | 0.02 | 0.007 |  |  |  |
| **Sclerostin** | **ln, mmol/L** | - | - | - |  |  |  | -0.07 | 0.05 | 0.2 |  |  |  |
| **DKK1** | **ln, mmol/L** | -0.18 | 0.13 | 0.2 |  |  |  | - | - | - |  |  |  |
| **Blood plts** | **log, ng/L** | -0.002 | 0.0003 | <0.0001 |  |  |  | 0.001 | 0.0002 | <0.0001 | 0.001 | 0.0002 | <0.0001 |
