# Supplementary material for: Circulating levels of sclerostin but not DKK1 associate with laboratory parameters of CKD-MBD
Source: PLoS One. 2017 May 11;12(5):e0176411. doi: 10.1371/journal.pone.0176411 (PMC5426702; doi:10.1371/journal.pone.0176411)
Supplement: S2 Table — (DOCX) [file pone.0176411.s003.docx]

**Supplementary Table 2**: Linear regression analysis with Ln sclerostin and ln DKK1as dependent variable in CKD patients on maintenance hemodialysis

|  |  | **Ln sclerostin** | | | | | | **Ln DKK1** | | | | | |
| --- | --- | --- | --- | --- | --- | --- | --- | --- | --- | --- | --- | --- | --- |
|  |  | **Univariate** | | | **Multivariate (R²=0.16)** | | | **Univariate** | | | **Multivariate (R²=0.14)** | | |
| **parameter** | **unit** | B | SE | p | β | SE | p | B | SE | p | B | SE | p |
| **Age** | **yr** | 0.006 | 0.003 | <0.05 |  |  |  | 0.0005 | 0.003 | 0.9 |  |  |  |
| **BMI** | **Kg/m²** | 0.006 | 0.01 | 0.6 |  |  |  | 0.01 | 0.01 | 0.3 |  |  |  |
| **Gender** | **M 0, F 1** | -0.3 | 0.09 | 0.002 | -0.3 | 0.09 | 0.001 | 0.14 | 0.1 | 0.2 |  |  |  |
| **Diabetes** | **No:0; Yes:1** | 0.2 | 0.1 | 0.07 |  |  |  | 0.004 | 0.12 | 1.0 |  |  |  |
| **diuresis** | **mL** | -0.0002 | 0.0007 | 0.03 |  |  |  | 0.00008 | 0.0008 | 0.4 |  |  |  |
| **Ca** | **mg/dL** | -0.03 | 0.06 | 0.6 |  |  |  | 0.2 | 0.07 | 0.006 | 0.2 | 0.08 | 0.008 |
| **Phos** | **mg/dL** | 0.07 | 0.04 | 0.03 | 0.09 | 0.03 | 0.008 | -0.02 | 0.04 | 0.5 |  |  |  |
| **Bicarbonate** | **mmol/L** | -0.05 | 0.02 | 0.001 |  |  |  | -0.03 | 0.02 | 0.1 |  |  |  |
| **25(OH)D** | **ln, ng/L** | 0.004 | 0.08 | 1 |  |  |  | -0.04 | 0.09 | 0.6 |  |  |  |
| **1.25(OH)2D** | **Ln, pg/L** | 0.08 | 0.1 | 0.5 |  |  |  | -0.14 | 0.13 | 0.3 |  |  |  |
| **N TACT PTH** | **ln, pg/mL** | -0.11 | 0.04 | 0.006 | -0.14 | 0.04 | 0.0004 | -0.09 | 0.04 | 0.05 |  |  |  |
| **CAP PTH** | **Ln, pg/mL** | -0.08 | 0.05 | 0.09 |  |  |  |  |  |  |  |  |  |
| **FGF23** | **ln, mg/dL** | 0.04 | 0.03 | 0.3 |  |  |  | 0.03 | 0.04 | 0.5 |  |  |  |
| **Sclerostin** | **ln, mmol/L** | - | - | - |  |  |  | 0.04 | 0.07 | 0.5 |  |  |  |
| **DKK1** | **ln, mmol/L** | 0.04 | 0.07 | 0.5 |  |  |  | - | - | - |  |  |  |
| **Blood plts** | **log, ng/L** | -0.001 | 0.0006 | 0.03 |  |  |  | 0.002 | 0.0007 | 0.004 | 0.002 | 0.0007 | 0.003 |
